# Editorial: Sedentary Behavior in Human Health and Disease

**DOI:** 10.3389/fphys.2017.00901

**Published:** 2017-11-07

**Authors:** Daniel P. Bailey

**Affiliations:** Institute for Sport and Physical Activity Research, School of Sport Science and Physical Activity, University of Bedfordshire, Bedford, United Kingdom

**Keywords:** sedentary behavior, sitting, physical activity, energy expenditure, breaks in sedentary time

Sedentary behavior, defined as any waking behavior characterized by an energy expenditure ≤1.5 metabolic equivalents (METs) while in a sitting or reclining posture, has become a recognized independent risk factor for a wide array of health outcomes (Biswas et al., 2015). Technological advancements in modern society have created environments that encourage engagement in sedentary behavior, making this a public health concern. This Research Topic brings together contributions from researchers to advance the sedentary behavior research agenda and consider the case for reducing and breaking up sedentary time in primary prevention and disease management contexts.

The dangers of sedentary behavior may be particularly relevant to older adults who exhibit the highest amounts of sedentary time and are vulnerable to the adverse health effects of aging (Harvey et al., 2013). In this topic, Virtuoso et al. investigated whether self-reported sitting time could be used as a discriminator of frailty in hospitalized older adults (aged ≥ 60 years). Total daily sitting time was identified as a predictor of frailty with cut-points of >257 min/day and >330 min/day being predictive of the presence of frailty for males and females, respectively. In a slightly younger sample (40–75 years), van der Velde contributed a cross-sectional analysis of 1,932 adults from The Maastricht Study. Using an objective measure of sedentary behavior, total sedentary time were associated with a shorter 6 min walk test and lower relative elbow extension strength. There were favorable associations between the number of breaks in sedentary time per day and timed chair rise stand test performance. However, these associations were relatively weak, whereas associations between physical function measures with total and higher-intensity physical activity were stronger. These studies suggest that although sedentary time may increase the risk of frailty and reduce physical function, regular engagement in physical activity may be more important for improving and maintaining physical function in an older population.

In another cross-sectional study, Sardinha et al. contributed findings that total sedentary time and the number of breaks in sedentary time were associated with metabolic health in Type 2 diabetes, independent of moderate-to-vigorous physical activity and cardiorespiratory fitness. However, adjusting for cardiorespiratory fitness attenuated the association between total sedentary time and all but one glycaemic indicator, whereas the number of breaks in sedentary time had a favorable association with several glycaemic indicators independent of cardiorespiratory fitness. This suggests that high levels of cardiorespiratory fitness may neutralize the harmful effects of total sedentary time, but not *prolonged* sedentary time, in Type 2 diabetes.

To complement the growing experimental evidence that supports a causal relationship between sedentary behavior and metabolic health, Altenburg et al. contributed a pilot study that explored the effects of six consecutive days of increased prolonged sedentary time in free-living conditions in physically active young adult males. An increase in postprandial C-peptide was observed despite only a relatively small and insignificant increase in interrupted and uninterrupted sedentary time. No changes in glucose or triglycerides were observed, which may have been due to the relatively small increase in sedentary time during the experimental period. The authors provide important recommendations to overcome this limitation in future free-living research, such as objectively evaluating participants' normal baseline physical activity and sedentary time to ensure there is opportunity for them to substantially change their sedentary behavior during the experimental period. In a similar contribution, Duvivier et al. effectively changed participants' free-living sedentary behavior to permit a valid comparison between four consecutive days of (a) increased sedentary time and (b) substituting ≥7 h/day of sitting with light walking and standing in overweight adults. This resulted in a mean 13.5 and 7.6 h/day of sedentary time in these respective conditions. Favorable changes in insulin sensitivity, C-peptide, lipids and diastolic blood pressure were observed, which the authors suggest were similar in magnitude to responses observed when adhering to the 150 min/week physical activity guidelines. This highlights the potential importance of substituting sitting with light activities to reduce cardiometabolic disease risk in at-risk populations.

Based on growing evidence that reducing sedentary time may improve health, it is important to identify effective and feasible interventions for at-risk groups, such as office workers. Koepp et al. contributed an evaluation of an under-the-table-leg-movement apparatus. This apparatus was used during seated computer work at a desk and significantly increased energy expenditure by 18% compared to a standard office chair. However, this was not as high as the 107 and 155% increase in response to walking at 1 and 2 mph, respectively. Standing has also been recommended as an intervention to reduce sedentary time, although the benefits to metabolic health are inconsistent (Benatti and Ried-Larsen, 2015). Miles-Chan and Dulloo contributed a review of the large inter-individual variability in the energy cost of standing and identify that the energy cost of steady-state standing posture maintenance is considerably lower than the 1.5 METs threshold. However, regular postural transitioning (sitting to standing) appears to increase energy expenditure considerably more and may be most beneficial for overweight and obese individuals due to an increased postural transition energy cost. Naik et al. investigated electromyography muscle activities around the knee during sit-to-stand and returning task in females wearing shoes with different heel heights. Muscle imbalance around the knee during these tasks increased with increasing heel height, which may contribute to fatigue and knee problems, such as osteoarthritis. This should be considered when prescribing regular posture transitions as an intervention.

This research topic contributes to the mounting evidence highlighting the importance of avoiding high amounts of sedentary time, which may help in formulating public health guidelines. However, intervention development must take into account the population for which it is intended to ensure the strategies used are effective and do not predispose individuals to other health risks.

## Author contributions

The author confirms being the sole contributor of this work and approved it for publication.

### Conflict of interest statement

The author declares that the research was conducted in the absence of any commercial or financial relationships that could be construed as a potential conflict of interest.
